# A description of patients with recurrence of Pulmonary Tuberculosis in a Tuberculosis Hospital, Ermelo

**DOI:** 10.4102/phcfm.v3i1.261

**Published:** 2011-11-01

**Authors:** Ubon S. Akpabio, Pierre JT. de Villiers

**Affiliations:** 1 Ermelo Provincial Hospital, Ermelo, Mpumalanga Province, South Africa; 2 Department of Interdisciplinary Health Sciences, Stellenbosch University, South Africa

## Abstract

**Background:**

Retreatment TB (tuberculosis) is a serious category of pulmonary TB with a treatment outcome that could include MDR-TB (multidrug resistant TB). In the Msukaligwa municipality of Mpumalanga Province, South Africa, the burden of TB is high with poor treatment outcome indicators, thus creating some preconditions for retreatment TB. Knowledge of the characteristics of the patients and related health system factors would help in designing interventions to improve the care for patients, the adherence to medication and the prevention of retreatment TB.

**Aim of the study:**

The aim was to describe the occurrence, characteristics and management outcome of retreatment pulmonary tuberculosis in patients in a TB hospital in Ermelo.

**Objectives:**

The specific objectives were to describe the socio-demographic, behavioural and clinical factors related to recurrence of TB in patients; to determine the contribution of defaulting treatment to recurrence of TB in the study population; to identify the prevalence of resistance to TB medication amongst patients with retreatment TB; and to identify treatment outcomes in patients who have been followed up for the duration of retreatment TB.

This study was set in the 58-bed TB hospital in Ermelo.

The study design was cross-sectional and descriptive, and the study population comprised of patients admitted with TB at the Ermelo TB hospital between 01 January 2005 and 31 December 2007. Data were collected from the patients’ medical records and the TB registers by using a predesigned form. Data were analysed with Microsoft Excel Spreadsheet at the Centre for Statistical Consultation at the University of Stellenbosch.

**Results:**

All of the 388 patient records with retreatment TB, which formed 19.6% of TB patients admitted between 2005 and 2007, were reviewed. This comprised 66% male patients with a mean age of 41.4, and 34% female patients with a mean age of 35.3. They were mostly unemployed, and 93% had a primary education; 43% were unmarried and 34% were married. Retreatment TB was diagnosed with sputum smear microscopy in 71%, with a *bacilli* load of 3+ in 45% of the patients. Almost three-quarters (74%) of the patients have been afflicted by TB, 1–3 years before the episode under study. Retreatment TB categories were: ‘after treatment completed’, 69%; ‘default’, 19%; ‘after cure, 8%, and ‘treatment failure’, 4%. The majority, that is, 98% (169/172) of patients tested, had a HIV-positive status; the median CD4 cell count was 106 cells/µL at the time of retreatment, and very few (5%) were on ART (antiretroviral drug treatment). Drug resistance to primary TB drugs was as follows: Rifampicin (16%), Isoniazid (29%), Ethambutol (19%), and Streptomycin (23%). The treatment outcomes for those for whom data were available were: ‘successful’ (49.1%), ‘death’ (23.8%); and ‘treatment default’ (22.9%). MDR-TB caused complications in 3.3% of the patients.

**Conclusion:**

The majority of the retreatment TB patients were male patients with an average age of 41, and unemployed. More than two-thirds of the patients had completed TB treatment previously, and defaulting treatment accounted for less than one-quarter of retreatment categories. The process of care was better in terms of the diagnosis of TB with sputum smear. Improvement in the documentation of key factors such as smoking, alcohol, drug use amongst patients and co-morbidity, as well as counselling and testing for HIV and provision of ARTs, is required. Treatment outcomes with regard to successful outcomes should be monitored and improved.

## Introduction

Tuberculosis (TB) is a major public health problem worldwide, and during 1993 the WHO (World Health Organisation)^1^ has proclaimed it a public health emergency. South Africa is one of the high-burden countries for TB with an excessive annual estimated incidence.^2^ The WHO global report on TB in the 2005-estimate, places the incidence of TB in South Africa at 245 cases (200–302) per 100 000 population per year, and prevalence at 344–718 cases per 100 000 population. It is also estimated that mortality caused by TB, varies between 47 and 107 deaths per 100 000 patients (71/100 000), and that the proportion of new adult TB patients of age 15–49 who are HIV-positive, is 58% (49% – 65%); new TB patients who are multidrug resistant are estimated at 1.8% (1.4% – 2.3%) and previously treated TB patients who were multidrug resistant in 2002, are estimated at 6.7% (5.5% – 8.1%).^2^

In the Mpumalanga province of South Africa, the burden of tuberculosis reflects the national trend. The incidence of TB in the Msukaligwa municipal area is estimated as the second highest in the district at about 450 per 100 000.^3^ The available health indicators for 2006 for tuberculosis in the Msukaligwa municipal area, indicate a serious problem with the following: a Cure Rate of 40%; the Smear Conversion at the end of the intensive phase, 35%; and the Defaulter Rate, 27.5%.^3^

Improper treatment and default on treatment could result in TB *bacilli* developing resistance to treatment with serious consequences for the patient and the general population. Patients who present with retreatment pulmonary TB represent a group that could eventually lead to the development of drug resistance. Understanding the characteristics of these patients will help in designing interventions that will prevent non-adherence to treatment and enhance a high cure rate for patients with TB in the health care system, as well as minimise the burden of retreatment pulmonary TB on health care facilities and the community.

### Setting

The setting of this study is a 58-bed TB hospital in Ermelo, Msukaligwa municipality, of the Gert Sibande district of Mpumalanga. It is one of the two specialised TB hospitals in the district with facilities for the admission of adult TB patients. Patients for admission are referred from the one regional, the five district hospitals, the primary health care clinics, and private doctors, in the Gert Sibande district.

All retreatment TB patients who are ill and live far away from the hospital are usually admitted for an initial period of 6–8 weeks. During this admission, sputa are collected at intervals specified in the National TB Treatment Guidelines and the retreatment regimen commences. Decisions on the continuation of treatment, sputum culture, drug sensitivity and treatment failure are made, depending on the sputum smear results at these periods. On discharge, patients can be reviewed monthly as outpatients in the hospital, or followed up at the nearest primary health care clinics in their area. All patients who choose to be followed up at the clinics are provided with referral letters for the clinic sister. At the clinic they are referred for supervision of treatment under the DOTS (Directly Observed Treatment, Short-course) programme and will visit the clinic on a monthly basis to collect their medication, until the treatment is completed.

Retreatment tuberculosis occurs after a person may, or may not, have completed the scheduled treatment course for TB. It can occur because of endogenous reactivation or exogenous re-infection.^4, 5^ In the South African National TB Control Program,^6^ various categories of retreatment TB have been identified. The rates of recurrence of TB has been found to vary in various studies from 0% to 14% in one study,^7^ whilst a study^8^ amongst South African miners gave a rate of 10.3 episodes per 100 person-years at risk during a follow-up period of 25.1 months. In yet another study,^9^ a rate of 18% was indicated. Recurrence was found to be associated with poor treatment adherence and occurred after successful treatment, as well as after default on treatment.

An episode of retreatment TB could be a consequence of a TB relapse or a reinfection with a new TB strain. This differentiation can be performed by using DNA fingerprinting techniques with *Mycobacterium* TB.^8, 9, 10, 11, 12, 13, 14^ Whether retreatment TB in a patient is due to reinfection or reactivation is not clinically distinguishable. The mechanisms that have been postulated as leading to the development retreatment TB in patients are either endogenous and true treatment failure or exogenous reinfection. In an area of low TB incidence and low HIV prevalence, studies have revealed that reinfection with a new M. tuberculosis strain is considered a rare cause of recurrent TB.^14, 15^ This contradicted the findings in a population-based retrospective case control study^16^ in Houston, USA, where reinfection with a new strain of M. Tuberculosis was the cause of recurrent TB.

In a setting of high TB and HIV prevalence respectively, as in South Africa,^2^ the relative contribution of reinfection or relapse to recurrent TB is different. A study^10^ amongst South African mine workers indicated that most recurrent TB was due to relapse within 6 months, with reinfection common in patients with HIV infection, compared to an earlier study in South Africa^12^ using DNA fingerprinting which provided evidence that reinfection was the cause of recurrence of tuberculosis.

Non-adherence to TB treatment resulting in retreatment TB has been extensively studied, particularly in countries with a high burden of TB disease. The factors responsible can be categorised under personal (age, gender, marital status),^17, 18, 19, 20, 21, 22, 23, 24^ socio-economic or behavioural (employment, income, alcohol use),^26, 27, 28, 29, 30, 31, 32^ co-morbid,^33, 34, 35, 36, 37, 38^ health system,^39, 40, 41^ as well as community and family related groups. The issues of support from family or friends during treatment could reflect community beliefs, attitudes and stigma attached to TB and has been found to play a crucial role in defaulting.^32^

### Significance of the study

The aim of the study was to describe the occurrence, associated factors and management outcomes of retreatment pulmonary tuberculosis in patients in the Ermelo TB hospital.

## Ethical considerations

Ethical approval was granted by the Committee for Human Research at the University of Stellenbosch and the Research Ethics Committee of the Department of Health, Mpumalanga.

## Methods

### Design and materials

The study design was descriptive. The study population comprised of *all* patients admitted with TB at the TB hospital in Ermelo over a 3-year study period. The sample for the actual study population comprised all patients aged 15 and older who have been diagnosed with retreatment TB and who have commenced with TB treatment between 01 January 2005 and 31 December 2007. These are patients who have been entered in the hospital-based TB register with medical records available in the hospital. Patients excluded were those with extra pulmonary TB, and patients with retreatment TB who started with treatment in 2004 and completed their treatment in 2005.

The required information was extracted from the TB register and patients’ medical records and entered into the data collection form that was used for each identified patient. The data collection form had three-digit serial numbers with no patient identifiers on it, but could be cross-referenced with the medical record if necessary. Information for analysis was derived from the data collection form and entered into the Microsoft Excel spreadsheet designed for the analysis.

The reliability of the data collected was assured by clearly defining the terms and concepts relevant to the study. For this, the case definitions, as contained in the South African National TB control programme guidelines, were used. This applied to definitions such as ‘retreatment TB’; ‘retreatment categories’; ‘treatment outcomes’ and ‘drug resistance’. To ensure the validity of the data collected, reference was made to each of the patients’ records to ensure that the information reflected in the TB register corresponded to the patient's medical record.

### Procedure

The data collection involved visits to the Ermelo TB hospital during the period of the data collection. The total number of patients treated over the study period was ascertained from the TB register and patients with retreatment TB were identified. Medical records of the patients identified in the TB register, were requested in order to extract detailed information about each patient, based on the data collection tool developed for this purpose.

### Analysing

The data were analysed with Microsoft Excel Spreadsheet at the Centre for Statistical Consultation, University of Stellenbosch. The data collection form served as a guide to design the spreadsheet on which data on each of the variables contained in the questionnaire were entered, and subsequently analysed. This was a descriptive study and therefore the data analysis expresses the prevalence of various factors associated with retreatment TB using summary statistics. The data are presented mainly in the form of a histogram chart and tables, reflecting the variables of interest to the study.

## Definitions

The diagnosis of tuberculosis was considered to be based on any of the following: microscopy-sputum smear and standard reporting of the *bacilli* load; clinical evaluation of the patient (history, symptoms and signs of illness, and a physical examination); and chest radiography interpretation. In addition, any patient with TB who was on treatment with Regimen 2 was considered to have retreatment TB and in the absence of any documentation this was taken as pulmonary TB. In order to facilitate the collection of the required data, all the definitions applied to the study were those defined in the South African TB Control Programme Practical Guidelines 2000,6 as well as the definitions in national TB register. The following are definitions that are relevant to this study.

### Retreatment TB Categories

**Retreatment TB after treatment completed:** the patient had completed the course of treatment but no proof of cure is provided by the sputum smear test, but the patient now smear positive.

**Retreatment TB after cure:** the patient was treated and became smear-negative on completion of the treatment and now smear positive.

**Retreatment after default or interruption:** the patient interrupted the initial treatment for 2 or more months. The patient may be smear-positive or smear-negative, but still has active TB according to clinical or radiological assessment.

**Retreatment after failure:** The patient is on treatment but remained smear-positive 5 months or later after commencing TB treatment.

### Retreatment TB Outcomes

**Cured:** the patient is smear-negative at, or 1 month prior, to completion of the treatment, and on at least one previous occasion.

**Completed:** the patient has completed the treatment course but there is no proof of cure from the sputum test, that is, no smear-negative result is available.

**Failure:** the patient on treatment remained or became smear-positive 7 months or later after commencing treatment.

**Death:** the patient died during the course of treatment (regardless of the cause of death).

**Default:** the patient interrupted the treatment for 2 or more months.

## Results

A total of 388 patients with retreatment TB were seen at the TB Hospital between 2005 and December 2007. These constituted 19.6% of the 1980 patients, in total, treated for TB during the period. There were 66.0% male and 34.0% female patients, with a resulting male: female ratio of 1.9 : 1. The mean age for the male patients was 41.4 years and 35.3 years for the female patients. For both sexes, the most affected age group was 35–44 for men, and 25–34 for women (Table 1). Ninety-three percent (93.0%) had primary education, 2.0% had high school education and 6.0% had no formal education; 74.7% were unemployed. Information on social habits revealed the following: smoking, alcohol and drug use was not known for 98% of the patients. With regard to marital status, 43.0% were unmarried; in 23.0% the marital status was not known; 34.0% were reported as married. The following data were available on the number of people in the same household, excluding the patient at the time of the episode of retreatment TB treatment: the majority (72.0%) of the patients lived in households with least 2 persons.

**TABLE 1:** Age and sex distribution of retreatment tuberculosis patients (*N* =388).

Age Group Years	Male patients		Female patients		Total patients	
		
	*n*	%	*n*	%	*n*	%
15–24	6	1.5	15	3.9	21	5.4
25–34	64	16.5	56	14.4	120	30.9
35–44	94	24.2	37	9.5	131	33.8
45–54	64	16.5	20	5.2	84	21.6
55–64	23		5	1.3	28	7.2
≥ 65	4	1.0	0	0.0	4	1.0

**Total**	**255**	**65.7**	**133**	**34.3**	**388**	**100**

*Source*: Authors’ Original data *N*, The total number of patients; *n*, Given as a means of number.

The majority (97.0%) of the patients had TB once in the past and 74.0% of these patients had TB in the past 1–3 years before the episode under study. With regard to the diagnostic process for TB, most of the TB (71.0%) was diagnosed by Sputum microscopy (Table 2). The retreatment TB categories at the time of diagnosis (Table 3), and information on the drug sensitivity testing pattern for the primary TB drugs (Table 4) have been reflected. Records were available for analysis for about 25.9% (100/386) of the patients. With regard to drug sensitivity, more patients (29.0%) were resistant to Isoniazid compared to 16.0% for Rifampicin. In terms of treatment outcome (Table 5), successful outcomes were 49.1% for ‘cured and completed treatment’; ‘failure’, 4.2%; ‘default’, 22.9% and ‘death’, 23.8%, amongst those who were followed up at the TB hospital. MDR-TB resulted from 3.3% (7/214) of the patients followed up in the hospital. The ‘Treatment Outcomes’ for the retreatment TB. MDR-TB is included in the Failures. It is a serious complication of retreatment TB and not a treatment outcome.

**TABLE 2:** Distribution of retreatment tuberculosis patients according to how tuberculosis was first diagnosed (*N* =388).

Main Diagnostic tool	*n*	%
Sputum AFB	276	71.1
Chest X-Ray	93	24.0
Others (Pleural fluid ADA)	19	4.9

*Source*: Authors’ Original data *N*, The total number of patients; *n*, Given as a means of number; AFB, Acid-fast *bacilli*; ADA, Adenosine deaminase.

**TABLE 3:** Distribution of Retreatment tuberculosis patients by retreatment tuberculosis category based on previous tuberculosis episode (*N* = 388).

Retreatment TB Category	*n*	%
After treatment completed	266	69
After cure	31	8
After default	75	19
After failure	16	4

*Source*: Authors’ Original data *N*, The total number of patients; *n*, Given as a means of number; TB, tuberculosis.

**TABLE 4:** Distribution of retreatment tuberculosis patients by drug sensitivity testing profile.

Drug	*N*	*n*†	*n*‡	Sensitive	%	Resistant	%
Rifampicin	**386**	286	100	84	84.0	16	16.0
Isoniazid	**387**	287	100	71	71.0	29	29.0
Ethambutol	**387**	288	99	80	80.8	19	19.1
Streptomycin	**387**	288	99	76	76.7	23	23.2

*Source*: Authors’ Original data *N*, The total number of patients; *n*, Given as a means of number.

† Number of patients not tested.

‡ Number of patients tested.

**TABLE 5:** Distribution of retreatment tuberculosis patients by treatment outcome for patients followed up at the hospital (*N* = 214*).

Treatment Outcome	*n*	%
Cured	19	8.9
Completed Treatment	86	40.2
Failure†	9	4.2
Defaulted	49	2.9
Death	51	23.8

*Source*: Authors’ Original data *N*, The total number of patients; *n*, Given as a means of number.

* (44.8% [174/388] patients were transferred to the clinics and records were not available for review).

† Includes patients with MDR-TB (*n* = 7), MDR-TB rate of 3.3%.

MDR, multidrug resistant.

## Discussion

Although the proportion of retreatment TB in this study may not estimate directly the extent of retreatment TB in the population, it, however, gives some indication of the burden of this problem on the health system within the context of the already high prevalence of TB and HIV diseases in the community. It reflects a fair measure of the load of TB patients that the health care workers and the institution have to cope with. Various studies^7, 8^ have given estimates of retreatment TB in the patient population. In a systematic review of long term efficacy of DOT regimes^7^, the rates of retreatment TB was found to vary from 0% to 14% after successful treatment whilst a study^8^ amongst South African miners found a rate of 10.3 episodes per 100 person-years at risk during a follow-up of patients for the median period of 25.1 months with most recurrence occurring within the first 6 months of follow-up of patients. This study found that 74% of patients had contracted TB, 1–3 years before the current episode, which compares with the Malawian^47^ study that found that 60% of patients had another episode of TB within the first 2 years of completing treatment.

This study has also revealed some personal factors associated with retreatment TB amongst the patients studied. Most of the patients with retreatment TB were men with a mean age of 41.4 years compared to women who were relatively younger at an average age of 35.3 years. The mean age of female patients was at least 6.1years less than that of male patients. Whilst there have not been consistent findings on the effect of gender and default from TB treatment, most studies had shown that male patients are more likely to default with TB treatment than female patients. This may partly explain the relative large proportion of male participants with retreatment TB in this study. The finding of this study with regard to male patients is similar to others that have been reported from studies in Turkey, Nigeria and India. In the Turkish study,^25^ the risk factors for non-successful treatment were determined to be retreatment patients older than 46, whilst in the Nigerian study,^18^ the 44.2% who defaulted on their treatment were older than 65, and in India,^20^ not completing the process of diagnosis of TB was higher in the age group older than 50 years.

The prevalence of behavioural and social factors like unemployment, smoking, alcohol and drug use in patients with retreatment TB has also emerged from the study. Seventy five per cent of the study population indicated that they were unemployed at the time of treatment. This is the only social factor that was found in the study and compares well with what has been reported in studies in other parts of the world like Nepal, Russia and Brazil^26, 27, 28^ as one of the reasons for defaulting on TB treatment, which resulted in retreatment TB. There were little data on smoking, alcohol and drug use as it was not documented in the records. This is an important omission in the process of care of these patients which should be remedied and it should be realised that there is some linkage between these social habits and retreatment TB.

**FIGURE 1:**
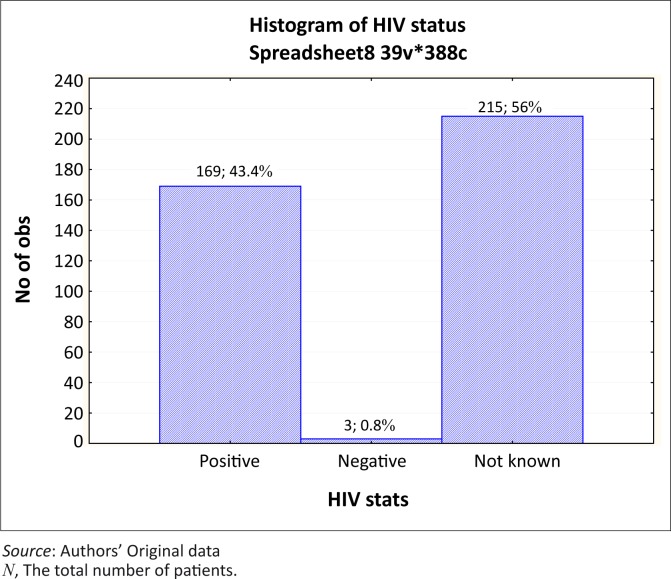
Distribution of retreatment TB patients by HIV status (*N* = 388).

There was some limited information on the HIV status of the patients with retreatment TB. Information on the HIV status was available for only 44.2% of the patients in this study (Figure 1). This low proportion could be related to the lack of capacity to offer voluntary counselling and testing to all patients and recording the results in the patients’ files. Amongst those who were tested, data indicate that 98.3% were HIV-positive. A previous study^34^ amongst South African miners has indicated that HIV infection was associated with significantly increased rates of recurrent TB with an incidence of 8.2 per 100 person-years in HIV-positive compared to 2.2 per 100 person-years in HIV-negative men. The need to test patients is recognised in the hospital; however, patients can decline after voluntary counselling.

Another HIV-related factor to the recurrence of TB is the level of immune-compromise as reflected by the CD4 counts. Information on this was available for a very small number of patients. Other studies^48, 49^ have showed that the risk of developing recurrent TB is increased as the immunity worsens with declining CD4 counts. In this study, the median CD4 count for those that were available was low at 106 cells/µL. The use of ART was found to be very limited in the study patient population. This was not unexpected as the roll out of ART in public sector was only initiated within the period of the study, hence most of those HIV-positive patients at that time did not have access to such services.

The study also revealed information on drug sensitivity patterns amongst patients. There was a large proportion of non-documentation relating to drug resistance and sensitivity to TB drugs used, as data was only available for 25.9% (100/386) of the patients studied. The low proportion of sensitivity testing reflects a serious lack of attention to this aspect of the care process for TB patients with regard to documentation in the patient files. The drug resistance to primary TB drugs varied from 16% for Rifampicin to 29% for Isoniazid. The level of resistance to any of the TB drugs is high when compared to other reported studies from Saudi Arabia^42^ and South Africa.^43, 44, 45, 46^ The findings of this study underlines the fact that resistance to any TB drug is significantly more probable in patients with retreatment pulmonary TB. The significance of this resistance lies in the potential for the emergence of multidrug resistance TB which is an important and serious unsuccessful outcome of retreatment TB.

The outcomes of retreatment TB in this study are only for those who were seen at the TB hospital and followed up on discharge. Those who defaulted on their treatment were those who were followed up as outpatients in the hospital after the initial phase of treatment. According to WHO,^2^ the targets of TB programs for new PTB patients are smear-positive detection rates of 70% and a cure rate of 85%. Whilst no specific targets for the outcomes have been set for retreatment TB, it would be logical to assume that they should not be less than for new TB cases. In terms of the smear-positive detection rate, the TB programme at the hospital has been managed competently. It achieved 71% compared with the WHO-set target of 70%, because the TB patients were mostly diagnosed with sputum smear microscopy. The treatment outcomes (cured and completed treatment) together were found to be 49.1%. This is much less than the 73% reported by WHO in its 2005 global review of TB programmes. Of great significance is the 3.3% MDR-TB found in this study. The MDR-TB is very expensive to treat and the longer treatment duration makes it difficult for patients to adhere to treatment. This proportion does not, however, derive from the estimated proportion of TB cases that were treated during the period which will lower the overall proportion. Amongst new TB cases in South Africa, previous studies had shown the MDR-TB rate to be relatively low at 1.6%, whilst that for retreatment TB was 6.6% (4.0%–13.9%).31 Compared to this, our figure of 3.3% for the retreatment TB cases is relatively small. This should, however, be seen against the setting that about 44% of the retreatment TB patients were transferred to other facilities for which information on outcomes was not available.

### Limitations of the study

The study is limited by its design which is mainly descriptive and retrospective in nature. The study relies on the previously collected data and information on the patient file for analysis. Some of the information was incomplete, thus making it difficult to have a complete perspective of the problem under study. In situations where the diagnosis of TB was based on the chest radiograph, this information was accepted as it was recorded and the study did not seek to look at the chest radiograph in question. The treatment outcomes of patients discharged from the TB hospital and followed up at the clinics were not ascertained because of limited resources available to the researcher to visit the clinics and to collect the data needed.

The study is further limited by the fact that there is no control or comparison group on which to make an in-depth analytical appraisal of the identified related factors of retreatment TB. Time and resources had not made this possible. This aspect should be explored in a future study.

### Recommendations

All TB patients (new or retreatment TB) should be offered voluntary counselling and testing for HIV, consistently. Those who are HIV-positive should be enrolled into ART programmes and monitored regularly to detect any deterioration in their immune status by regular CD4 measurements and necessary intervention should be started.

Improve process of care by appropriate and complete documentation in the patient file of all potential factors impacting on retreatment TB at the time of admission into the treatment programme and any new developments reviewed and updated as situations develop in the patients over the period of care. This will provide sufficient data on which the burden of the problem could be understood and allow dominant factors identified to be addressed with relevant interventions. Strengthening the institutional capacity to do this will be required in terms of personnel and other resources for monitoring and reporting on outcomes for retreatment TB patients.

Further researches should be undertaken that involve data collection from the patients, family and treatment partners directly, using quantitative and qualitative approaches to allow for quantitative analyses of factors and test associations with retreatment TB.

## Conclusion

This study has confirmed that some factors related to retreatment TB are similar to what were obtained elsewhere. In our situation majority of patients had completed TB treatment previously and default from treatment accounted for less than one quarter of the patients. There was a large proportion of non-documentation relating to drug resistance and sensitivity to TB drugs which reflects a serious lack of attention to this aspect of the care process for TB patients. The treatment outcomes (cured and completed treatment) together, were found to be much less than the reported target for TB programmes. For those tested for HIV, they were overwhelmingly positive. The quality of care for these patients needs to be improved by paying attention and documenting these factors. This study opens up areas of further research in our setting to test associations and prominence of some of the identified factors.
